# What do we know about redox status? A review in neonates of mothers with preeclampsia

**DOI:** 10.1080/07853890.2026.2688414

**Published:** 2026-08-26

**Authors:** Juan Carlos Avilés-Garcia, María Fernanda Aguilar-Dueñas, Guillermo Ceballos, Yessica Dorin Torres-Ramos

**Affiliations:** ^a^ Departamento de Inmuniobioquímica, Instituto Nacional de Perinatología Isidro Espinosa de los Reyes, Ciudad de México, México; ^b^ Escuela Superior de Medicina, Instituto Politécnico Nacional, Ciudad de México, México; ^c^ Escuela Nacional de Ciencias Biológicas, Instituto Politécnico Nacional, Ciudad de México, México; ^d^ Laboratorio de Investigación Integral Cardiometabólica, Escuela Superior de Medicina, Instituto Politécnico Nacional, Ciudad de México, México

**Keywords:** Oxidative stress, preeclampsia, neonatal outcomes, lipid oxidation, antioxidant enzymes, non-enzymatic antioxidants

## Abstract

**Background:**

Preeclampsia (PE) is a leading cause of maternal and perinatal morbimortality worldwide, driven by oxidative stress from an imbalance between reactive oxygen and nitrogen species and antioxidant defenses. Neonates born to mothers with PE face an oxidized intrauterine environment, yet their antioxidant response and potential therapeutic strategies remain insufficiently characterized.

**Methods:**

A literature review was conducted searching PubMed, Redalyc, CORE, and Google Scholar for English-language papers using keywords including oxidative stress, preeclampsia, neonatal outcomes, antioxidant defence, NRF2, polysulfides, and antioxidant supplementation.

**Result:**

Neonates from mothers with PE exhibit elevated lipid peroxidation markers (conjugated dienes, lipohydroperoxides, malondialdehyde) yet show compensatory increases in total antioxidant capacity. The neonate’s antioxidant defense system is summarized, including its enzymatic components superoxide dismutase (SOD), glutathione peroxidase (GPx), catalase (CAT), and non-enzymatic factors, with a particular focus on novel reducing molecules known as polysulfides. Investigation into persulfides and polysulfides is not merely supplementary but essential, as these emerging redox species represent a promising and potentially superior therapeutic frontier for mitigating oxidative damage. The article also discusses contradictory findings on the impact of antioxidant interventions for PE, highlighting the inherent challenges of clinical trials in this population. We examine the potential therapeutic role of the transcription factor NRF2, alongside concerns about possible side effects.

**Conclusions:**

Current antioxidant supplementation for PE has proven largely ineffective or harmful. Persulfides and polysulfides emerge as a potentially superior antioxidant frontier for neonatal protection, warranting dedicated investigation. Well-designed clinical trials are urgently needed to evaluate the safety and efficacy of novel antioxidant strategies for improving neonatal outcomes in PE.

## KEY MESSAGES

- Preeclampsia remains a leading cause of maternal and neonatal morbidity, driven by oxidative stress from excess reactive oxygen and nitrogen species that damage placental and fetal tissues.
- Evidence suggests that neonates from mothers with preeclampsia develop compensatory antioxidant responses, yet these are insufficient to prevent oxidative damage completely.
- Current antioxidant supplementation trials (e.g., vitamins C and E) show inconsistent or adverse outcomes, underscoring the need for cautious policy on antioxidant use in pregnancy.
- Emerging evidence identifies polysulfides as novel sulfur-based molecules with potent antioxidant and NRF2-activating properties, representing a promising but underexplored therapeutic avenue.

## GRAPHICAL ABSTRACT

**Figure:**
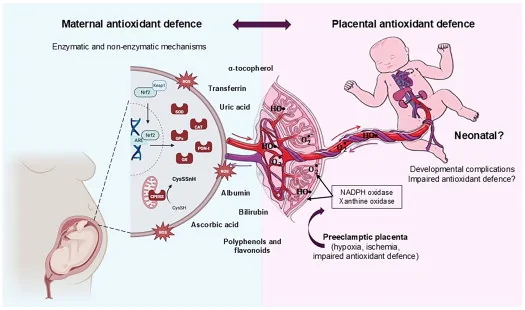

## 1. Introduction

Preeclampsia (PE) is a severe pregnancy complication characterized by high blood pressure and multiorgan dysfunction, posing risks to both the mother and the foetus due to the complexity itself related to placental hypoxia and inflammation processes. This multisystemic condition represents a noteworthy public health concern, as it stands among the primary causes of maternal and perinatal morbimortality worldwide [1,2]. Observations have indicated that oxidative stress (OS), stemming from a disparity between ROS production and antioxidant defence mechanisms, is intertwined with the development and progression of PE. Oxidative stress involves the interplay of diverse molecules governing cellular electronegativity against oxidizing or reducing molecules (antioxidants). The homeostasis of these oxidation-reduction reactions in cells and tissues is pivotal for sustaining their vitality. This equilibrium is governed by a reactive species system (oxygen (O_2_), superoxide anion (O_2_^•−^), hydrogen peroxide (H_2_O_2_), hydroxyl radical (HO^•^), peroxide nitrite (ONOO^−^), among others) alongside with enzymes and proteins that curtail the reactivity of those species (reduced/oxidized glutathione (GSH/GSSG), superoxide dismutase (SOD), catalase (CAT), glutathione peroxidase (GPx), Paraoxonase (PON-I), collectively constituting and contributing to the redox state [3]. Various authors describe oxidative stress as a “biochemical process where an imbalance arises between the generation of free radicals and the capacity of the organism to counteract them through antioxidant mechanisms”. The erythrocyte is an excellent oxidative stress biomarker due to its proficient reducing capability. These blood cells transport oxygen to body tissues and are consistently exposed to oxidative stress due to the high content of unsaturated lipids in their membranes [4]. Concurrently, they can be deemed excellent models for investigating PE and intrauterine effects when subjected to the characteristic oxidative stress of this condition [5]. In an inherently hypoxic intrauterine environment, neonates need to develop efficient antioxidant systems [6]. Usually, these antioxidant systems counteract oxidative damage effectively, and evidence indicates they experience less oxidative deterioration than their mothers, potentially due to an overactivated antioxidant system [7]. Thus, comprehending these mechanisms has profound significance for a vast understanding of the pathologies and underlying mechanisms to counteract oxidative stress-induced disorders. Furthermore, studying oxidative stress in the perinatal population is pertinent, as manifold researchers have associated links between oxidative stress and adverse perinatal outcomes [8–10]. This review examines the impact of PE in terms of oxidative stress and its homeostatic regulators in PE. Understanding the connections between maternal and neonatal OS is crucial for advancing knowledge of perinatal complications and potential therapeutic approaches. Hence, we will explore the impact of oxidative stress in the perinatal intrauterine environment based on current literature, encompassing lipid peroxidation, alterations in erythrocyte membranes, therapeutic exogenous antioxidant strategies, novel antioxidant molecules named “polysulfides”, and the involvement of the Nuclear Factor Erythroid 2-Related Factor 2 (NRF2) transcription factor in this process. By evaluating the available evidence, this review provides a comprehensive understanding of antioxidant interventions, their potential benefits, and limitations, aiming to enhance neonatal outcomes in the hospital setting within the context of PE.

## 2. Materials and methods

Papers and/or abstracts published in English were searched using PubMed, Redalyc, COnnecting Repositories (CORE), and Google Scholar between July and September 2025, including the following keywords: oxidative stress, preeclampsia, neonatal outcomes, antioxidant defence, NRF2, enzymatic defence, antioxidant supplementation, oxidative stress biomarkers, and polysulfides.

## 3. Are reactive oxygen species necessary?

Reactive oxygen species (ROS) include not only oxygen radicals but also certain non-radical oxygen derivatives (O_2_), such as hydrogen peroxide (H_2_O_2_), hypochlorous acid (HOCl), and ozone (O_3_). Therefore, while all oxygen radicals are ROS, not all ROS are oxygen radicals [11]. ROS play a crucial role in maintaining the homeostatic environment and promoting cell survival. They also serve a significant physiological function in defence mechanisms, rendering them crucial elements for cellular adaptation and survival [12].

In terms of concepts,” reactive” is a relative term; oxygen (O_2_) and water (H_2_O) exhibit high selectivity in their reactions with biological molecules, leaving most untouched, whereas the hydroxyl radical (HO^•^) indiscriminately attacks its surroundings. Therefore, some authors prefer the term “oxygen-derived species” over ROS. Another commonly used collective term is “oxidants”; however, Zhang et al. mention that because the superoxide anion (O_2_^•−^), hydrogen peroxide (H_2_O_2_), and some other ROS can act as both oxidizing and reducing agents in different systems, employing this term is not advisable. Moreover, the term “reactive species” has been expanded to encompass reactive species of nitrogen, chlorine, bromine, and sulfur [13].

The release of ROS mediated by NADPH oxidases (NOX family) activities leads to eradicating microorganisms in macrophages and neutrophils, thus serving as an inflammatory mediator. NOX genes produce transmembrane proteins responsible for the electron transport chain across biological membranes, reducing molecular oxygen to superoxide anion, which occurs under non-pathological conditions [14].

Vascular NOX is generated to maintain cardiovascular health by synthesizing endothelium-derived relaxing factor (EDRF) and nitric oxide (NO), which help regulate blood pressure, an essential factor for overall well-being [15]. It also inhibits platelet aggregation and leukocyte adhesion to the endothelium [16], among other functions. Furthermore, both NO and H_2_O_2_ are involved in controlling vasodilatory responses in coronary arteries [17]. However, high ROS levels reduce the bioavailability of EDRF and NO.

Another example of ROS involvement in function is the kidneys. ROS produced through NOX3 activity regulates renal function by controlling Na^+^ transport. Moreover, oxygen-derived species enhance NaCl absorption in the Henle loop, resulting in the modulation of Na1/H1 exchange [18].

In the context of viral infections, the generation of ROS limits influenza virus spread by causing death in infected cells through signalling pathways. ROS also act as second messengers to trigger redox-sensitive transcription factors, eliciting subsequent immune and inflammatory responses that eventually can cause damage. Reduced ROS generation through transcription factor depletion or malfunctioning NOX enzymes potentially favours pulmonary influenza infection by promoting the development of professional phagocyte populations [14,19]. ROS act as “second messengers” in certain situations; their optimal concentrations activate a range of cell signal transduction pathways, facilitating the actions of growth factors like cytokines and calcium signalling. Beyond its role as an essential metabolic intermediate, hydrogen peroxide (H_2_O_2_) acts as a central signaling molecule that senses, modulates, and regulates redox processes within the cell. They can trigger a cascade of proteins through specific oxidations, resulting in cellular metabolic responses. This metabolic response can influence cell proliferation, survival, or death, depending on the subsequent pathways activated (homeostatic, pathological, or protective) [20].

Cell exposure to reactive species triggers a series of signalling pathways of NRF2, nuclear factor kappa (NF-kB), and mitogen-activated protein kinases (MAPKs), which can induce adaptive or inflammatory cellular responses [21]. Their binding initiates protein phosphorylation, setting off these signalling cascades. Concerning the concept of “second messengers,” Halliwell et al. mention that the more appropriate term is “secondary messengers,” which modify the size or duration of another signal (i.e. receptor binding-dependent phosphorylation) [22].

## 4. Do we really need antioxidants?

The term “antioxidant” is often used to refer to an inhibitor of lipid peroxidation [23]. A broader definition includes “any substance that, when present in low concentrations compared to an oxidizable substrate, significantly delays, or prevents the oxidation of the said substrate”. Notably, the term “oxidizable substrate” encompasses nearly all molecules found *in vivo*. This definition underscores both the “damage” and “source” aspects used in examining antioxidant action [24]. Table 1 illustrates the evolution of the antioxidant concept over time, along with its respective modifications and specifications.

**Table 1.** Evolution of the antioxidant concept.

Year	Reference	Evolution of the Antioxidant Concept
1989	[22]	An antioxidant is a molecule capable of inhibiting the oxidation of other molecules. Its definition is “any substance that delays, prevents or eliminates oxidative damage to a target molecule.”
1992	[25]	Antioxidants protect the human body from free radicals and the effects of ROS. They slow the progression of many chronic diseases and lipid peroxidation.
1997	[24]	An antioxidant is a substance that directly eliminates ROS or indirectly regulates antioxidant defences or inhibits ROS production. Antioxidant compounds can remove radicals and extend lifespan by slowing the process of lipid peroxidation.
2006	[26]	Activity refers to the constant reaction rate between a specific antioxidant and an oxidant. Capacity measures the amount of a specific free radical removed by a sample.
2011	[27]	Antioxidants retard lipid peroxidation and the formation of lipid secondary products.
2013	[28]	Antioxidants help reduce amino acid oxidation and protein oxidation, as well as the interaction of carbonyl lipid derivatives with proteins, leading to protein alteration.
2020	[29]	Antioxidant activity is based on the availability of electrons to neutralize free radicals. Furthermore, it is related to the number and nature of hydroxylation patterns on the aromatic ring.

Over time, the understanding of the antioxidant role has expanded to include neutralising free radicals. Presently, antioxidants are recognized not only for countering oxidative stress by donating electrons to stabilize free radicals but also for involvement in cell signalling pathways, modulation of gene expression, and protection of biomolecules against various forms of oxidative stress. It is widely known that endogenous antioxidant defences must operate as a balanced and coordinated system. A reactive species elimination system must collaborate with an enzymatic defence system [22] to be effective. In this regard, it has been demonstrated that liposomes containing catalase (CAT) and superoxide dismutase (SOD) provide more effective protection against O_2_ toxicity compared to liposomes containing only one of these enzymes, compared to liposomes containing only SOD or CAT, which showed lower levels of protection, providing further evidence for the cooperation between defence systems [30,31].

Therefore, ROS and antioxidants are necessary in an appropriate balance for proper cellular function to remain effective. This broader and more complex perspective on antioxidants has emerged thanks to enriching studies, which will be discussed later, that identify various molecules with antioxidant capacity and therapeutic potential, such as the novel molecules called polysulfides [32].

## 5. Polysulfides and persulfides

Persulfides and polysulfides are molecules that are based on the sulfane sulfur atom. These can be classified according to their chemical nature as: inorganic (RSSH = hydropersulfides; RSSnH = hydropolysulfides, *n* > 2) and organic (RSSR = persulfides, the “R” group represents a biomolecule, for example: protein-RSS; RSSnR; *n* > 2 = polysulfides). In this review, the term “persulfides/polysulfides” will be used to collectively refer to the chemical species “RSSH/RSSnH”, depending on the functional group with which they are bound [33,34]. Sulfane sulfur is defined as a reactive form formed by two sulfur atoms with oxidation number “-1”, bonded to each other. This configuration gives it high reactivity, especially in its exposed atom, allowing it to be incorporated into various proteins [35].

Previously, persulfides and polysulfides were studied together with hydrogen sulfide (H_2_S) due to limited knowledge about their own characteristics. However, the growing interest in their biochemical properties has given them an essential role in research, closely related to the study of H_2_S as a gas transmitter [32]. RSSH/RSSnH sythesis is carry out by secondary participation of enzymes such as cystathionine-β-synthase (CBS), cystathionine-γ-lyase (CSE) and 3-mercaptopyruvate sulfurtransferase (3-MST) has been proposed, which are involved in the sythesis of persulfides and polysulfides through the oxidation of H_2_S [36,37], nonetheless, Abidin et al. [38] compared triple-knockout mouse animal models of these three enzymes (CBS, CSE, and 3-MST) and mice deficient in mitochondrial cysteinyl-tRNA synthetase (CAR2), where they elucidated that the latter enzyme is mainly liable for the activity of cysteine persulfur synthase (CPERS) and consequently the persulfide/polysulfide synthesis pathway. Both H_2_S and persulfides/polysulfides contrast in their biogenesis; they also do so in their structural properties, since RSSH/RSSnH are based on sulfane sulfur atoms linked to thiol group of cysteine [39].

Chemical attributes of RSSH/RSSnH are often compared to thiols/disulfide (RSH/RS-SH), probably because of redox equilibrium these species establish *in vivo* [36]. There are many arguments to propose that RSSH/RSSH are better reducing agents than thiols: under physiological pH conditions, they are in favored imbalance towards deprotonated perthiolate anion species (RSS-), while thiols prevail in their protonated form [40]; have improved nucleophilic and reducing activity, it is associated that there is an alpha effect between electrons of the lone pair in sulfane sulfur atoms, and a greater stabilization of RSSH against oxidation (RSS•, pertyl radical) since the unpaired electron is not located in the exposed sulfur atom, an effect that is not possible with thiyl radical (RS•), respectively [41,42].

These chemical characteristics of persulfides/polysulfides have led to their study as antioxidant molecules, capable of impeding oxidative damage to different biomolecules. The antioxidant activity of different polysulfides, such as dialkyl polysulfides (RSnR, *n* = 1–4) [43], disodium tetrasulfide (Na_2_S_4_), and diallyl tetrasulfide [44] have been reported, highlighting favorable results in inhibition of lipohydroperoxides formation compared to other molecules such as glutathione and Trolox.

Delving into these reducing properties, Griffiths et al. [45] demonstrated in a mouse model of ischemia-reperfusion cardiac injury that cysteine trisulfide (Cys-SSS-Cys) administration -an enzyme or nonenzyme precursor to cysteine persulfur (CysSSH)- produces a cytoprotective effect, significantly reducing malondialdehyde (MDA) and 4-hydroxynonenal (4-HNE) levels compared with a control group. In addition to their antioxidant properties, RSSH/RSSnH upregulate antioxidant responses, such as the Keap1-NRF2 pathway [46,47], cellular senescence mechanisms [48,49], and anti-inflammatory responses [50,51].

Given multiple roles played by these novel molecules, basic and biomedical research has focused on evaluating their pathophysiological role. In Table 2, we summarize what has been reported by various authors on RSSH/RSSnH or similar (H_2_S, GSSSH, among others) measurements in some pathologies.

**Table 2.** Summary of studies reporting alterations in sulfane sulfur–containing species, including persulfides (RSSH), polysulfides (RSSnH), and hydrogen sulfide (H_2_S), across various pathological conditions.

Pathology	Sulfane sulfur source	Study type	Tissue	Findings	Reference
Portal hypertension	H_2_S	Case-control	Human plasma	H_2_S levels were lower than healthy controls.	Wang, et al. [52].
Diabetic retinopathy	Cysteine persulfides (CysSSH), oxidized glutathione trisulfide (GSSSG) and cystine	Case-control	Aqueous and vitreous humors	CysSSH, GSSSG, and cystine were elevated in the aqueous humor; CysSSH and Cys were elevated in the vitreous.	Kunikata, et al. [53].
Asthma–chronic obstructive pulmonary disease overlap (ACO)	RSSH and RSSnH	Case-control	Human sputum	Levels of reactive RSSH and RSSnH were significantly decreased in sputum from the patients with ACO.	Kyogoku, et al. [54].
Alzheimer and related dementias (ADRD)	H_2_S, bond sulfide pools	Case-control	Human plasma	Levels of total H_2_S were increased in ADRD versus controls, total plasma sulfide pools was the strongest indicator of ADRD, related between cognitive dysfunction and white matter lesion volume.	Disbrow, et al. [55].

Previously, some pathologies have been described where RSSH/RSSnH or sulfur molecules are involved; however, what is known about these molecules in neonates? It has been described under the Developmental Origins of Health and Disease (DOHaD) concept that renal programming is related to an important role of inorganic and organic sulfur compounds [56] and *H*∼2∼S in regulating placentation, vascular adaptation and fetal development, in parallel, an imbalance of these molecules and oxidative damage, is related to various conditions during pregnancy (hypertension, preeclampsia, gestational diabetes) [57–59]. However, the antioxidant implications of persulfides/polysulfides in neonates of mothers with PE remain unknown, but interventions with this approach are lacking.

## 6. Preeclampsia

Preeclampsia (PE) is defined as new-onset hypertension and new-onset target organ damage after 20 weeks of gestation [1]. This clinical syndrome initiates with abnormal trophoblast invasion before clinical manifestations of the disease become apparent. The pathogenesis of PE is rooted in early placental development, beginning with inadequate trophoblast invasion and spiral artery remodeling. In a healthy pregnancy, trophoblasts infiltrate the decidua, transforming the spiral arteries to secure robust, autonomous blood flow to the placenta. However, in PE, this process is disrupted as trophoblasts do not fully differentiate into an endothelial phenotype. The consequent incomplete vascular remodeling results in placental ischemia. This ischemic environment triggers the upregulation of key anti-angiogenic factors, notably soluble fms-like tyrosine kinase-1 (sFlt-1) and soluble endoglin (sEng). sFlt-1 is pivotal in maternal and fetal pathology; it sequesters vascular endothelial growth factor (VEGF) and placental growth factor (PlGF), depleting these critical mediators of endothelial health, particularly in vulnerable tissues like the kidneys and liver. Simultaneously, sEng impairs cellular signaling by binding to and inhibiting transforming growth factor-beta (TGF-β), a molecule essential for endothelial cell migration and proliferation. The combined action of sFlt-1 and sEng precipitates extensive maternal endothelial dysfunction. This systemic injury manifests as vasoconstriction, oxidative stress, and microemboli, which ultimately drive the multi-organ failure characteristic of PE [60].

## 7. The relationship between oxidative stress and preeclampsia

Oxidative Stress (OS) is the result of the discrepancy between the generation and neutralization of reactive species, which can lead to the accumulation of intermediate products of Reactive Oxygen Species (ROS) and Reactive Nitrogen Species (RNS), both of which have crucial biological functions in appropriate amounts. However, these imbalances can induce oxidative stress [5]. OS is a detrimental process that negatively affects cell membrane structure, lipids, proteins, lipoproteins, and deoxyribonucleic acid (DNA). Immoderate production of hydroxyl radicals (HO^•^) and peroxynitrite (ONOO^-^) leads to lipid peroxidation, damaging cell membranes and lipoproteins. This reaction yields cytotoxic and mutagenic compounds, such as conjugated dienes (DC), lipohydroperoxides (LPH), and malondialdehyde (MDA). Lipid peroxidation rapidly increases, affecting numerous lipid molecules. Consequently, proteins also undergo structural modifications that can lead to the loss or impairment of their respective enzymatic activity [61]. An example of damage through lipid peroxidation is seen in erythrocytes, which are vulnerable to lipoperoxidation due to their role in transporting oxygen to the developing foetus, thus experiencing membrane alterations through OS. These membrane changes include decreased fluidity and increased rigidity, compromising erythrocyte functionality and their ability to traverse the microvasculature. Consequently, erythrocytes become more susceptible to oxidative stress, potentially leading to lipid peroxidation. This process, involving the oxidation of polyunsaturated fatty acids in cell membranes, generates MDA and other by-products as markers of oxidative damage [27].

Evidence from animal models indicates that oral administration of high doses of peroxidised lipids causes damage, such as cardiac impairment in rats, “fatty liver” in rats and rabbits, and immune system damage in mice. Occasionally, the toxicity may not be attributed to lipid peroxides but to protein carbonylation formed through their breakdown in the stomach [24]. Oxidized lipids may be similar to H_2_O_2_; at low levels, they can stimulate cell proliferation, while at higher levels, they can inhibit it, and even at higher concentrations, they can induce apoptosis and necrosis. These events occur in atherosclerosis [24].

Apart from lipid peroxidation, prolonged OS causes DNA damage, forming 8-hydroxy-2′-deoxyguanosine (8-OHdG). The latter can mispair with thymidine instead of cytosine, considered a deleterious DNA lesion responsible for mutagenesis. Furthermore, oxidative DNA damage may lead to loss of epigenetic information, possibly because of impaired active methylation of the CpG island in gene promoter regions. 8-OHdG has been studied as a tissue biomarker of oxidative stress [5].

Considering all these consequences, it can be inferred that oxidative stress can induce various acute and chronic diseases and accelerate cellular ageing processes. It is hypothesized that oxidative stress could be a likely risk factor for PE onset due to the primary inability of antioxidant capacity to offset free radicals and the increase in oxidative stress product levels [61]. Multiple studies have focused on the role of oxidative stress in women with PE, demonstrating that placental tissue from PE cases produces higher levels of superoxide compared to average pregnant women [5]. Thus, a close relationship emerges between neonatal effects and growth within an intrauterine environment with oxidative imbalance.

The role of oxidative stress in pregnant patients with PE is explained through various mechanisms. During the ischemic phase of PE, oxygen and nutrient deficiency lead to hypoxanthine accumulation, calcium release, xanthine oxidase activation, and induction of proinflammatory cytokines. The reperfusion phase is characterized by a marked increase in oxidants like NO, ONOO^-^, and O_2_•^-^, which originate from multiple sources. These include the hypoxanthine/xanthine oxidase system, mitochondria, iNOS (NOS2), and NOX enzymes present in endothelial cells, infiltrating neutrophils, and local tissue cells [62].

The oxidants produced through these pathways initiate a vicious cycle in PE. Oxidative stress plays a cyclical role in PE, as increased inflammation generates higher levels of ROS, leading to a constant cycle of impairment unless underlying processes (antioxidants) countering this progression are sufficient. Alteration in blood flow through the placenta causes severe hypoxia and release of preeclamptic factors into the systemic circulation. These factors promote vasoconstriction and hypertension, as well as the activation of neutrophils and platelets in the maternal vascular system [63].

Regarding oxidative stress-derived products in PE pregnancies, an increase in lipid damage in lipoproteins, elevated levels of conjugated dienes, lipohydroperoxides, and malondialdehyde has been reported [64]. Furthermore, some authors [63,65] have noted that maternal and umbilical cord blood in PE groups, compared to the average group, show higher levels of Maternal Total Antioxidant State (M-TAS). Concerning neonates, oxidative damage presents differently; a study conducted on neonates born to mothers with PE [64] did not show evident protein oxidation damage and, conversely, indicated an increase in total antioxidant capacity. This suggests a well-developed defence system in neonates born to mothers with PE.

Oxidative Stress (OS) is the result of the discrepancy between the generation and neutralization of reactive species, which can lead to the accumulation of intermediate products of Reactive Oxygen Species (ROS) and Reactive Nitrogen Species (RNS), both of which have crucial biological functions in appropriate amounts. However, these imbalances can induce oxidative stress [5]. OS is a detrimental process that negatively affects cell membrane structure, lipids, proteins, lipoproteins, and deoxyribonucleic acid (DNA). Immoderate production of hydroxyl radicals (HO^•^) and peroxynitrite (ONOO^-^) leads to lipid peroxidation, damaging cell membranes and lipoproteins. This reaction yields cytotoxic and mutagenic compounds, such as conjugated dienes (DC), lipohydroperoxides (LPH), and malondialdehyde (MDA). Lipid peroxidation rapidly increases, affecting numerous lipid molecules. Consequently, proteins also undergo structural modifications that can lead to the loss or impairment of their respective enzymatic activity [66]. An example of damage through lipid peroxidation is seen in erythrocytes, which are vulnerable to lipoperoxidation due to their role in transporting oxygen to the developing foetus, thus experiencing membrane alterations through OS. These membrane changes include decreased fluidity and increased rigidity, compromising erythrocyte functionality and their ability to traverse the microvasculature. Consequently, erythrocytes become more susceptible to oxidative stress, potentially leading to lipid peroxidation. This process, involving the oxidation of polyunsaturated fatty acids in cell membranes, generates MDA and other by-products as markers of oxidative damage [24].

Evidence from animal models indicates that oral administration of high doses of peroxidised lipids causes damage, such as cardiac impairment in rats, “fatty liver” in rats and rabbits, and immune system damage in mice. Occasionally, the toxicity may not be attributed to lipid peroxides but to protein carbonylation formed through their breakdown in the stomach [22]. Oxidized lipids may be similar to H_2_O_2_; at low levels, they can stimulate cell proliferation, while at higher levels, they can inhibit it, and even at higher concentrations, they can induce apoptosis and necrosis. These events occur in atherosclerosis [22].

Apart from lipid peroxidation, prolonged OS causes DNA damage, forming 8-hydroxy-2′-deoxyguanosine (8-OHdG). The latter can mispair with thymidine instead of cytosine, considered a deleterious DNA lesion responsible for mutagenesis. Furthermore, oxidative DNA damage may lead to loss of epigenetic information, possibly because of impaired active methylation of the CpG island in gene promoter regions. 8-OHdG has been studied as a tissue biomarker of oxidative stress [5].

Considering all these consequences, it can be inferred that oxidative stress can induce various acute and chronic diseases and accelerate cellular ageing processes. It is hypothesized that oxidative stress could be a likely risk factor for PE onset due to the primary inability of antioxidant capacity to offset free radicals and the increase in oxidative stress product levels [66]. Multiple studies have focused on the role of oxidative stress in women with PE, demonstrating that placental tissue from PE cases produces higher levels of superoxide compared to average pregnant women [5]. Thus, a close relationship emerges between neonatal effects and growth within an intrauterine environment with oxidative imbalance.

The role of oxidative stress in pregnant patients with PE is explained through various mechanisms. During the ischemic phase of PE, oxygen and nutrient deficiency lead to hypoxanthine accumulation, calcium release, xanthine oxidase activation, and induction of proinflammatory cytokines. The reperfusion phase is characterized by a marked increase in oxidants like NO, ONOO^-^, and O_2_•^-^, which originate from multiple sources. These include the hypoxanthine/xanthine oxidase system, mitochondria, iNOS (NOS2), and NOX enzymes present in endothelial cells, infiltrating neutrophils, and local tissue cells [63].

The oxidants produced through these pathways initiate a vicious cycle in PE. Oxidative stress plays a cyclical role in PE, as increased inflammation generates higher levels of ROS, leading to a constant cycle of impairment unless underlying processes (antioxidants) countering this progression are sufficient. Alteration in blood flow through the placenta causes severe hypoxia and release of preeclamptic factors into the systemic circulation. These factors promote vasoconstriction and hypertension, as well as the activation of neutrophils and platelets in the maternal vascular system [67].

Regarding oxidative stress-derived products in PE pregnancies, an increase in lipid damage in lipoproteins, elevated levels of conjugated dienes, lipohydroperoxides, and malondialdehyde has been reported [68]. Furthermore, some authors [61,67] have noted that maternal and umbilical cord blood in PE groups, compared to the average group, show higher levels of Maternal Total Antioxidant State (M-TAS). Concerning neonates, oxidative damage presents differently; a study conducted on neonates born to mothers with PE [68] did not show evident protein oxidation damage and, conversely, indicated an increase in total antioxidant capacity. This suggests a well-developed defence system in neonates born to mothers with PE.

## 8. What do We know about the effect of preeclampsia on neonates?

Having established the connection between PE and OS, and understanding that OS can impact signalling pathways such as NRF2 [62], NF-kB [21], and Stress-Activated Protein Kinases (MAPK), affecting multiple biological processes including protein modification, inflammation promotion, apoptosis induction, and autophagy dysregulation [63], we will now focus on the effect of PE and an oxidized intrauterine environment on offspring.

In a broader context, the stress theory suggests that foetal OS leads to increased lipid peroxidation and, more essentially, gene expression modifications that result in adverse perinatal programming. The susceptibility of an individual foetus will rely on both the timing of metabolic programming during gestation and the genetic susceptibility of the foetus [64]. Hence, neonates born to mothers with PE, facing an oxidized intrauterine environment, are expected to develop adverse perinatal effects.

In the quest for tools to assess the impact of oxidative stress in neonates, specific biomarkers have been studied. These markers, such as levels of free radicals and lipid peroxidation, have demonstrated a correlation with clinical outcomes in neonates born to mothers with PE. Measuring them provides valuable insight into oxidative stress status and its association with neonatal complications [65]. This is why we will use them as parameters to review in data collection.

Several authors [67,69–71] have identified higher oxidative stress markers in maternal blood and umbilical cord blood in PE groups compared to the control group. It has recently been proposed that oxidative stress might play a fundamental role in pregnancy-related developmental processes that predispose individuals to insulin resistance, glucose dysregulation, and obesity [72].

A study conducted in India concluded that neonates from mothers with mild and severe PE had a higher frequency of morbidities such as respiratory distress syndrome (RDS) and early-onset sepsis. Multivariable logistic regression analyses revealed a strong association between lower M-TAS levels and poor neuromotor outcomes in children at one year of age [67]. Furthermore, they inferred a significant correlation between maternal protein carbonylation and early neonatal outcomes like death and respiratory distress syndrome.

**Figure 1.**
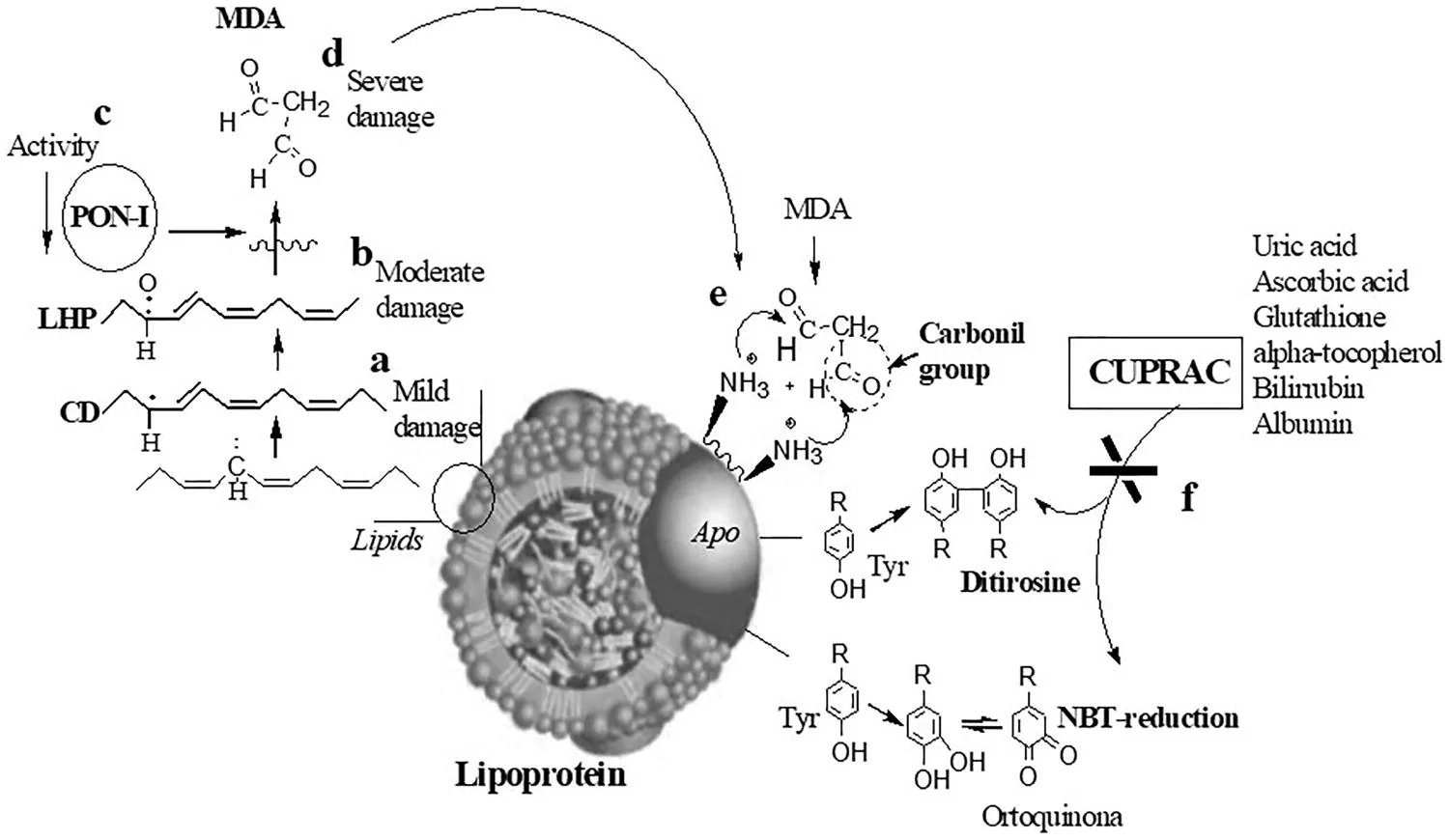
Mechanisms of oxidative damage to LDL-c and HDL-c lipoproteins in newborns of women with PE. Both lipoproteins experience lipid damage; molecular products resulting from lipoperoxidation include conjugated dienes (mild damage) (a), lipohydroperoxides (moderate damage) (b), and malondialdehyde (severe damage) (c). Lipohydroperoxides can be hydrolysed by the enzyme PON-I. When the activity of this enzyme decreases (d), malondialdehyde levels increase. Additionally, this process can damage proteins by forming adducts, inducing carbonyl groups (f). Other indicators of protein oxidation include orthoquinone formation. These can reduce the NBT compound and/or form dityrosines (e). In neonates of women with PE, these latter two damage mechanisms do not occur, likely due to increased overall systemic antioxidant capacity. Non-protein antioxidants such as uric acid, ascorbic acid, glutathione, and alpha-tocopherol, and protein antioxidants such as bilirubin and albumin, protect against damage (g). **Note:** Image taken from Leon-Reyes G et al. Oxidative profiles of LDL and HDL isolated from women with PE. Lipids Health Dis. 2017 May 16;16(1):90.

In a study examining oxidative damage to LDL-c and HDL-c lipoproteins in neonates born to mothers with PE, Leon Reyes et al. describe a spectrum of molecular products resulting from lipoperoxidation–-conjugated dienes (mild damage), lipohydroperoxides (moderate damage), and malondialdehyde (severe damage)–-which are generated by hydroxyl radical (HO•) attack on lipids [68] (Figure 1). The same study found that these neonates exhibited no indicators of protein oxidation or dityrosine formation, a protection the authors attribute to the neonates’ robust antioxidant defenses, encompassing both non-protein antioxidants (uric acid, alpha-tocopherol, ascorbic acid, glutathione) and protein antioxidants (bilirubin, albumin) [7] (Figure 1).

In the same sense, in a case-control study, higher levels of MDA and oxidative stress were observed in umbilical cord blood and were linked with an increased risk of low Apgar scores, admission to the Neonatal Intensive Care Unit (NICU), and assisted ventilation for the newborn [69].

We summarise several systematic reviews that investigated the effects of an intrauterine environment with PE on neonates. Each review provides an overview of included studies, evaluated parameters, and relevant neonatal health and well-being outcomes (Table 3). Newborns from mothers with PE are particularly vulnerable to oxidative stress due to their underdeveloped antioxidant defence systems [5]. Therefore, comprehending the mechanisms underlying oxidative stress in PE and understanding the maternal and neonatal antioxidant status is crucial for identifying promising therapeutic interventions [61].

**Table 3.** Description of key effects on offspring from an intrauterine environment with PE.

Year	Author	Information
1985	[73]	Newborns from mothers with PE had elevated fibrinogen, reduced activity of II, V, and VII, and decreased antigen II.
2000	[74]	Over 3 years, only 2.39% of babies were born alive after pregnancies with PE and PE with severity data. PE was associated with a 5% reduction in birth weight. In severe PE, the reduction was 12%, and in early-onset disease, birth weight was 23% lower than expected.
2004	[75]	There is a negative impact on offspring exposed to PE, and a negative impact on cognitive function throughout life was found compared to the average population.
2008	[76]	Being born to a mother with PE is linked to higher rates of infantile respiratory distress syndrome, intraventricular haemorrhage, sepsis, bronchopulmonary dysplasia, and neurological developmental disability in childhood.
2009	[77]	According to PE severity, hypertension is increased risk in adults exposed to maternal PE with severity data.
2015	[78]	Being born to a mother with PE has a higher rate of foetal death; 5.2 per 1000 foetal deaths in women with PE versus 3.6 per 1000 in women with uncomplicated pregnancies. In early-onset PE, the risk of foetal death is even seven times higher than in normotensive pregnancies.
2015	[79]	Typical anti-hypertensive therapy in PE can generally increase the risk of intrauterine foetal growth restriction, persistent foetal bradycardia, and hypotension.
2016	[80]	PE is not limited to pregnancy but increases cardiovascular risk in later stages of the offspring’s life.
2016	[81]	There is a cardiovascular risk in offspring after intrauterine exposure to PE. Most studies found higher systolic blood pressure in the age range of 9 to 17 years, while others found higher diastolic blood pressure, and some found both.
2019	[82]	Children of preeclamptic pregnancies show risk factors for CVD during childhood and young adulthood.
2022	[83]	Severe forms of respiratory distress syndrome (RDS) were twice as frequent in the group of new-borns exposed to early-onset PE compared to the control group. Neutropenia and thrombocytopenia were more common in premature new-borns exposed to PE. The severity of PE correlates with haematological changes.

## 9. Antioxidant defence systems in neonates

Antioxidant defence systems, encompassing the pair of enzymatic and non-enzymatic antioxidants, collaborate synergistically to counteract oxidative stress. Enzymatic antioxidants such as SOD, GPx, and CAT work to neutralize ROS and safeguard against oxidative damage. Non-enzymatic antioxidants, including vitamins C and E, glutathione, and uric acid, eliminate free radicals and inhibit lipid peroxidation [22]. Concerning the mother, antioxidants, concretely CAT, SOD, reduced glutathione (GSH), and oxidised glutathione (GSSG), are present throughout average pregnancies, serving to protect against natural oxidative stress during gestation [80,81]. However, in cases of PE, antioxidant defence systems may become overwhelmed, resulting in a disparity between ROS production and antioxidant capacity [5]. Nonetheless, the neonatal response to this condition remains relatively underdefined.

During the initial 10 weeks of gestation, human placentas exhibit low antioxidant levels due to reduced O_2_ tension (<20 mmHg). As maternal intraplacental circulation becomes fully established between weeks 11 and 14, oxygen levels rise to around 50 mmHg, triggering a surge in reactive oxygen species (ROS) production, accompanied by enhanced synthesis of CuZnSOD, GPx, and MnSOD [83].

Thus, during gestation, the developing foetus requires highly efficient antioxidant systems to counterbalance the naturally hypoxic intrauterine environment, which is crucial for proper foetal development. However, after birth, exposure to hyperoxia can generate reactive oxygen and nitrogen species, leading to oxidative stress and free radical production. Newborns face a sudden transition from a warm, fluid-filled environment to cooler air inhalation, exposing their lungs to much higher O_2_ concentrations than experienced in utero. Severe hypoxia often occurs during the actual birth, making the eventual exposure to 21% of O_2_ even more challenging for the neonate [6].

Moreover, birth asphyxia can trigger a significant increase in oxidative stress, prompting the neonate to mature its antioxidant defence system. Preparations for birth occur during the late stages of intrauterine development and involve increased levels of antioxidants like extracellular superoxide dismutase (EC-SOD), other SODs, GSH, catalase, GPx, and peroxiredoxins in the lung. The transcription factor FOX2 plays a pivotal role in control of these events and surfactant synthesis. While some antioxidants, such as MnSOD and CuZnSOD, appear early in gestation, others (e.g. catalase, thioredoxin) do not emerge until the last four weeks of gestation [83].

Regarding the antioxidant system neonates face during the intrauterine period, a study evaluated a total of 100 PE and 50 healthy pregnant women. Placental levels of CAT, SOD, and a positive correlation between GSH and GSSG, as well as IL-6, were described in pregnant women with PE, compared to the reference group. Correlations were identified between placental GSH levels and birth weight, head circumference, chest circumference, and gestational age at birth. PE-derived placentas exhibited elevated concentrations of antioxidant enzymes and thiols, potentially acting as a compensatory system against oxidative stress. The only biomarker that demonstrated a positive correlation with favorable perinatal outcomes was placental glutathione (GSH) concentration, indicating its critical role in fetal health and maternal protection in PE [69]. The role of antioxidants in neonates born to mothers with PE becomes crucial to counteract the impact of oxidative stress and free radicals in the postnatal circumstances. However, substantial cohort and case-control studies on neonatal antioxidant function remain lacking.

## 10. NRF2 transcription factor

As mentioned earlier, there’s a lack of data concerning the actual functioning of antioxidant systems in neonatal environments. Nevertheless, it is known that in adults, the NRF2 transcription factor plays a crucial role in managing antioxidant response elements and inducing the expression of various antioxidant and detoxifying enzymes [63]. The activity of the transcription factor NRF2 may be compromised in placentas of mothers diagnosed with PE, resulting in reduced expression of antioxidant enzymes, hypoxia, and inadequate syncytiotrophoblast invasion. Restoring NRF2 activity holds promise as a therapeutic strategy to mitigate oxidative stress and improve neonatal and maternal outcomes in PE. Experimental models exploring NRF2 activation through NRF2 inducers or phytochemicals have demonstrated potential in enhancing antioxidant defences [63,84]. The antioxidant effect of extracts from tea, cocoa, and many dietary plants is linked to their ability to activate NRF2 and boost antioxidant enzymes. The mechanism involves the oxidation of these compounds into electrophilic quinones that bind to Kelch-like ECH-associated protein 1 (KEAP1) cysteines [63].

This mechanism is not unique to dietary extracts but forms the basis for a major class of antioxidant therapies. The therapeutic potential of NRF2 activators lies in their ability to bolster the body’s endogenous antioxidant defences, thereby mitigating disease processes. A central strategy in developing antioxidant therapies involves upregulating protective enzymes through the NRF2 signalling pathway. The efficacy of many small-molecule compounds, such as polyphenols, is largely attributable to this mechanism [85]. These NRF2 activators can be categorized into five distinct classes based on how they regulate the pathway: 1) KEAP1 modifiers: These agents directly target the KEAP1 protein, which normally flags NRF2 for proteasomal degradation, by forming adducts with its sensor cysteines; 2) βTrCP Interaction Disruptors: This class interferes with the βTrCP protein, another negative regulator of NRF2, by disrupting the GSK3β-mediated phosphorylation that recruits βTrCP; 3) KEAP1 Sequestrators: Exemplified by p62, these molecules bind and sequester KEAP1, preventing it from interacting with NRF2. 4) De Novo NRF2 Synthesis Inducers: These strategies promote the production of new NRF2 protein that can accumulate because the available KEAP1 is already occupied or inactivated. 5) BACH1 Inhibitors: This category reduces the suppression of NRF2 by inhibiting the transcriptional repressor BACH1, either by blocking its synthesis or promoting its degradation [86]. The preclinical promise of NRF2 activators has propelled notable progress in pharmaceutical development. However, this progress is tempered by inconsistent clinical outcomes and a critical lack of human data confirming that these compounds successfully upregulate antioxidant defences in target organs, leaving their true therapeutic efficacy in need of further validation [63].

The therapeutic use of NRF2 activators is complicated by their potential for off-target effects and paradoxical outcomes. A primary issue is their lack of specificity; these compounds can influence multiple signalling pathways beyond the intended NRF2 axis. A well-characterized example is sulforaphane, which exerts anti-inflammatory effects by inhibiting NF-kB and inflammasome activation, and can halt cell proliferation *via* suppression of the PI3K-AKT and MAPK-ERK pathways [87,88]. Furthermore, because NRF2 activation is not tissue-specific, its systemic induction can lead to unintended side effects. This systemic activity underlies a significant oncological concern: while NRF2 activation may offer chemopreventive benefits, it can also support tumour progression by enhancing the antioxidant capacity of cancer cells, thereby fostering resistance to chemotherapy [63].

## 11. Exogenous antioxidants as a therapeutic method

Thus far, the therapeutic use of antioxidant molecules has been discouraging, mostly due to overly optimistic and erroneous suppositions about how antioxidants function. For instance, the direct elimination of the hydroxyl radical (HO^•^) is impractical, but effective prevention of its formation can be achieved by reducing hydrogen peroxide (H_2_O_2_). It is a common misconception that small-molecule antioxidants can efficiently intercept superoxide (O_2_^•−^) or H_2_O_2_ in a cellular context. Their uptake is ineffective because antioxidant enzymes far outpace them in reaction speed, establishing enzymatic pathways as the dominant protective mechanism. However, in extracellular fluids where antioxidant enzymes are absent, synthetic mimics of superoxide dismutase and catalase can indeed remove O_2_^•−^ and H_2_O_2_ (but not HO^•^) [89]. A contrasting perspective from other authors [90] is that antioxidant therapy, encompassing supplements, flavonoids, and polyphenols, can effectively reverse oxidative damage when integrated with moderate aerobic exercise.

Current clinical research is investigating a diverse array of antioxidant therapeutic approaches. These strategies can be broadly categorized by their mechanism: 1) some aim to scavenge reactive oxygen species at the source, such as by inhibiting NOX enzymes or bolstering mitochondrial defences; 2) others focus on neutralizing specific oxidants—for instance, removing superoxide (O_2_•^-^) to prevent peroxynitrite (ONOO^-^) formation, or eliminating hydrogen peroxide (H_2_O_2_) before it can generate more damaging hydroxyl radicals (HO•) or hypohalous acids (HOX); 3) involves enhancing the body’s intrinsic antioxidant systems, either by providing glutathione (GSH) precursors or by upregulating protective enzymes through pathways like NRF2 activation. Additional avenues include dietary antioxidant supplementation and the targeted inhibition of dysfunctional redox signaling [63].

In the context of PE, until recently, data supporting the success of vitamin C and vitamin E supplementation for its prevention were limited. Published trials focused on antioxidant use in pregnant women at high risk of developing PE. A randomized study involved 283 women at high PE risk based on abnormal uterine artery Doppler waveforms or disease history. Those receiving antioxidant therapy exhibited a lower PE rate than controls (8 per cent vs. 17 per cent), along with a significant furtherance in endothelial and placental function markers (ratio of plasminogen activator inhibitor type 1 to type 2) [91].

Similarly, studies have indicated that supplementing high-risk women with 400 IU/day of alpha-tocopherol plus 1 g/day of vitamin C reduces the PE incidence, suggesting that, occasionally, low nutritional antioxidant levels could serve as risk factors. However, as further studies have been conducted, the effects appear less impressive. Vitamin C supplements also protected certain women against premature rupture of chorion-amnion membranes during pregnancy, potentially due to vitamin C deficiency affecting collagen synthesis [67].

Nevertheless, a systematic review and meta-analysis conducted by Conde-Agudelo et al. encompassed nine trials involving a total of 19,810 women. It was concluded that, overall, there was no significant difference in the risk of PE between the vitamin and placebo groups. Similar results were acquired when subgroup analyses were restricted to women at high or low/moderate PE risk. Also, the study found that vitamin C and E supplementation led to a higher risk of gestational hypertension and premature placental detachment, alongside a lower risk of placental abruption. However, there were no other statistically significant differences in adverse outcomes for either the mother or the fetus/newborn between the treatment and placebo groups. Consequently, supplementation during pregnancy with vitamin C and E do not prevent PE [92].

Given the limited evidence of benefit and potential for harm, supplementary antioxidant therapy for PE prevention should not be prescribed as part of routine practice. Yet, a potent *in vitro* antioxidant doesn’t necessarily guarantee *in vivo* efficacy. For instance, it may be rapidly excreted, metabolized into an inactive product, or fail to reach the correct action site.

N-acetylcysteine (NAC) is regarded as one of the most well-established antioxidants for therapeutic purposes. NAC exerts its antioxidant effect mainly by replenishing glutathione, a process initiated when it is deacetylated to cysteine within cells. NAC can also reduce plasma cysteine conjugates. NAC’s failure to exert therapeutic effects could stem from oxidative stress being a secondary donor to the disease under study [93].

The therapeutic application of other antioxidants has likewise been complicated by inconsistent results. For example, although the SOD-based drug orgotein was never approved for human use after its development in the 1970s, its anti-inflammatory properties have been the subject of clinical trials. One double-blind, placebo-controlled study showed it was safe and effective at preventing radiotherapy side effects like cystitis and proctitis in bladder cancer patients. Nonetheless, in another clinical study, orgotein revealed non-therapeutic effects on radiation response or acute radiation reactions, causing adverse reactions like marked subcutaneous infiltration and localized injection site redness in some patients. Currently, orgotein is utilized as an anti-inflammatory agent in animals [94].

On the other hand, the use of antioxidants is known to have adverse effects on neonates, with reports indicating that mothers supplemented with vitamins C and E during pregnancies complicated by PE or gestational hypertension were more likely to give birth to infants with low birth weight [95,96], small size for gestational age [97,98], preterm birth <37 weeks [97,98], foetal death [95,99], stillbirth [95,99,100], neonatal death [95,99,100], perinatal mortality [95,99,100], congenital malformation [97,100,101], admission to the NICU [95,98,102], respiratory distress syndrome [95,99,100], necrotizing enterocolitis [95,99,100], neonatal sepsis [97,100], retinopathy of prematurity [95,99,100], intraventricular haemorrhage (any grade) [95,99,100], intraventricular haemorrhage grade III/IV [95,102], periventricular leukomalacia [95,99,100], neonatal seizures [95,99,100], need for mechanical ventilation [96,98], and chronic lung disease [97,103].

## 12. Discussion

According to Jakubczyk et al. [11], various species are involved in the generation of reactive oxygen species (ROS). Although attempts have been made to isolate these species for experimental study, there is a possibility that interactions between species may modify the studied system behaviour. Considering this approach, it is proposed not to attribute oxidative stress to a single specific species, “the whole is more than the sum of its parts.” Understanding the relationships and connections between the components of a system is essential for a comprehensive understanding of its function and significance. However, this species diversity may pose limitations for future research, as existing studies primarily quantify *in vitro* enzymatic activities and may be influenced by numerous confounding factors among study subjects.

Some studies have suggested a relation between preeclampsia and an increase in the production of ROS and RNS, unstable and highly reactive molecules that can cause cellular damage. Antioxidants could be crucial in counteracting the excess ROS and protecting cells and tissues from oxidative damage. However, as noted by Zhao and colleagues, the therapeutic use of antioxidants in various pathologies related to oxidative stress has been disappointing.

Contradictory results have emerged from studies involving pregnant women at high risk of developing PE. One study demonstrated only an 11 per cent difference in the likelihood of developing PE compared to the control group [91], which remains controversial. Systematic reviews and meta-analyses affirm that no significant differences exist between patients who took antioxidants and those who took a placebo [92]. Conversely, it has been observed that women supplemented with vitamins C and E had a higher risk of developing gestational hypertension [92,98,101], severe gestational hypertension [95,103], and premature rupture of membranes [99,101].

Long-term effects of antioxidant supplementation during the neonatal period have also yielded adverse outcomes. Researchers studied the long-term effects of antioxidant interventions during the neonatal period and explored whether early antioxidant supplementation had lasting benefits on birth outcomes. The results were conclusive in revealing multiple adverse effects due to maternal antioxidant administration during pregnancy [92]. It is intriguing to note that the observed neonatal outcomes are similar to those of an intrauterine environment with PE, such as admission to the NICU and assisted ventilation for the new-born [69], stillbirths, foetal death [75,104], reduced birth weight [104,105], respiratory distress syndrome, and the need for assisted ventilation [73]. This suggests that neonatal outcomes are likely a consequence of the intrauterine environment damaged by PE or gestational hypertension, rather than solely a result of antioxidant therapy during pregnancy, thus demonstrating the null effect of antioxidants in reducing neonatal adverse effects.

The proposal of an efficient biomarker to assess a panoramic view of the association between oxidative stress and PE, as well as its enzymatic antioxidant activity, could not only lead to diagnosis and monitoring of PE but also be a timely therapeutic target identification to prevent neonatal consequences, thereby developing more specific and effective treatments. One of the most controversial issues of our time is the study of the NRF2 transcription factor, which induces the expression of multiple antioxidant enzymes. As noted by Forman and Zhang in 2021 [63], it is one of the most promising experimental models, showing potential as a therapeutic strategy to mitigate oxidative stress and alleviate multiple oxidative stress-related pathologies. However, it has also been demonstrated that while stimulating and activating NRF2 and inducing antioxidant enzymes, other signalling pathways are simultaneously activated, disrupting critical biological processes. Moreover, its effect is not limited to a single cell, and *in vitro*, results have shown that NRF2 can also promote the development of cancer cells and resistance to chemotherapy drugs.

Polysulfides, particularly allyl sulfides, found in garlic and other plants, have a direct relationship with the activation of the transcription factor NRF2 [106]. The activation of NRF2 by polysulfides is a significant mechanism for cellular protection against oxidative stress [107]. NRF2 activation occurs when a compound such as polysulfide breaks the bond between NRF2 and its repressor, the KEAP1 protein [108]. Under homeostatic conditions, KEAP1 keeps NRF2 in the cytoplasm and marks it for degradation. However, polysulfides react with the cysteine residues of KEAP1, modifying their structure. This modification prevents KEAP1 from binding to NRF2, thereby stabilizing NRF2 and facilitating its translocation to the nucleus. Once in the nucleus, NRF2 attach to DNA sequences known as antioxidant response elements (AREs), activating the transcription of genes that encode antioxidant and detoxifying enzymes. In short, polysulfides act as chemoprotective agents by releasing NRF2 from KEAP1’s control, allowing the cell to activate its antioxidant defense system [109,110].

Several authors suggest that the most promising therapeutic approach is to block the production of damaging oxidants, rather than attempting to increase levels of antioxidant enzymes and their cofactors. This could open the way for new therapeutic strategies in the neonatal hospital environment. Understanding how antioxidants interact and how their effects can be controlled would be valuable in the context of PE and other medical conditions related to oxidative stress.

## 13. Limitations and future directions

While the potential role of antioxidants in newborns of mothers with PE seems promising, several limitations and challenges exist. Firstly, clinical trials investigating antioxidant interventions in this specific population are limited, making it challenging to establish concrete recommendations. Additionally, the optimal timing, dosages, and methods of antioxidant administration remain uncertain. Future research should focus on well-designed randomized controlled trials to accurately assess the efficacy and safety of antioxidant interventions for improving neonatal outcomes in PE. Furthermore, exploring innovative approaches, such as integrating interdisciplinary methods, may shed light on innovative theoretical frameworks and analytical strategies for studying oxidative stress and antioxidant therapies in PE.

The potential for NRF2 activation to accelerate tumor progression and confer chemotherapy resistance raises significant safety concerns. Consequently, some researchers advise against the systemic use of NRF2 activators in vulnerable populations, particularly patients receiving chemotherapeutic treatment. Although other adverse effects from sustained NRF2 activation are less documented, research efforts are focused on mitigating risks through novel drug design. Proposed strategies aim to enhance specificity, such as creating non-electrophilic compounds and prodrugs that are selectively activated within microenvironments of elevated oxidative stress [111].

The effectiveness of antioxidant defenses is constrained by the significant contribution of oxidative stress to PE and its impact on the offspring. When oxidative stress plays a secondary role in disease, as in the case of PE and its effects on the neonate, preventing oxidative stress may not have a significant impact on disease development. In fact, this is a major reason why antioxidants exert little or no effect on the pathology, even when they clearly increase antioxidant defence and decrease oxidative stress markers. This boundary is perhaps the most important factor that is often overlooked when considering antioxidant defences in clinical trials.

## 14. Conclusion

Antioxidants play a crucial role in relieving oxidative stress and protecting newborns of mothers with PE. Damage induced by oxidative stress, including alterations in erythrocyte membrane and lipid peroxidation, can be alleviated by activating antioxidant defence systems, mainly the NRF2 transcription factor. Furthermore, it is critical to note that oxidative stress cannot be attributed to a single reactive oxygen or nitrogen species but involves a diversity of species. This poses challenges in researching and evaluating antioxidant interventions, as numerous factors can influence study outcomes. We consider it highly relevant to study the role of persulfides and polysulfides, as these are emerging redox sulfur species with antioxidant capacity and regulatory functions superior to those of many antioxidants already tested in clinical trials. Despite growing evidence of their role in adult diseases, their antioxidant potential in neonatal outcomes, particularly in the context of preeclampsia, remains largely unexplored, representing a crucial avenue for future research.

Ultimately, while antioxidants play an essential role in cellular protection and the potential mitigation of oxidative stress, their therapeutic use is not as straightforward as anticipated. Further research, especially well-designed clinical trials, is needed to assess their efficacy and safety more accurately in the context of PE and other medical conditions related to oxidative stress. While limitations and challenges exist, future research should prioritize well-designed clinical trials to promote a better awareness of the role of antioxidants and develop evidence-based strategies to improve neonatal outcomes in PE. Ultimately, comprehending the complex interaction between oxidative damage and antioxidants in this context will open the way for effective therapeutic interventions. Furthermore, understanding the onset of oxidative damage and evaluating its mechanism will inform the proposal of a specific antioxidant to reduce observed oxidative damage.

## Data Availability

Data sharing is not applicable to this article as no data were created or analysed in this research.
